# Multicomponent Exercise Program Reduces Frailty and Inflammatory Biomarkers and Improves Physical Performance in Community-Dwelling Older Adults: A Randomized Controlled Trial

**DOI:** 10.3390/ijerph17113760

**Published:** 2020-05-26

**Authors:** Uratcha Sadjapong, Supachai Yodkeeree, Somporn Sungkarat, Penprapa Siviroj

**Affiliations:** 1 Department of Community Medicine, Faculty of Medicine, Chiang Mai University, Chiang Mai 50200, Thailand; uratcha.s@gmail.com; 2 Department of Biochemistry, Faculty of Medicine, Chiang Mai University, Chiang Mai 50200, Thailand; yodkeelee@hotmail.com; 3 Department of Physical Therapy, Faculty of Associated Medical Sciences, Chiang Mai University, Chiang Mai 50200, Thailand; ponlaor@gmail.com

**Keywords:** multicomponent exercise, frailty, muscle strength, balance, biomarkers, quality of life

## Abstract

The efficacy of exercise to reverse frailty in the aging population has not been extensively investigated. This study aimed to investigate the effectiveness of a multicomponent exercise program (MCEP) on frailty, physical performance (handgrip strength, Berg Balance Scale (BBS), Timed Up and Go test (TUG), and VO_2_Max), blood biomarkers (Interleukin-6 (IL-6) and C-reactive protein (CRP)) in frail older adults. A randomized controlled trial using an allocation concealment method, included 64 older adults (77.78 ± 7.24 years), were divided into two parallel groups using block randomization: an MCEP group (*n* = 32) and a control group (*n* = 32). The combined center- and home-based MCEP training consisted of chair aerobic, resistance, and balance, which was carried out 3 days per week for 24 weeks. A mixed model repeated measure ANOVA demonstrated significant interaction effects of group x time for BBS, TUG and frailty scores (*p* < 0.001). Additionally, the post-hoc analysis revealed that the MCEP group showed significantly improved BBS, TUG, and frailty scores (*p* < 0.01), at both 12- and 24-weeks. When compared with controls at 12-weeks, the MCEP group decreased IL-6 and CRP levels (*p* < 0.05). The combined center- and home-based MCEP were effective in reversing frailty to pre-frailty and improving physical performance especially balance in the older population.

## 1. Introduction

There is an increase in the aging population in Thailand as there are developed and improved health care services and medical technology. In 2001 it was acknowledged that Thailand was starting to have an aging population with 7% of people aged 65 years and over, and is expected to be an aged society in 2023, with 14% of the population in this group [1,2]. Aging is frequently associated with a progressive decline in the ability to resist stress, damage, disease, and physical function [3]. The physical status of the elderly is generally classed as vulnerable to poor health outcomes. Thus, frailty is a well-known physical vulnerability status that is increasing [4].

Frailty is a biological syndrome associated with age defined as decreasing in the biological functional reserve and resistance to stressors’ physical status, resulting from accumulative declines in multiple physiological functions and increased adverse health outcomes as fall risk, hospitalization, disability, and death [5,6]. Fried et al. [4] defined five criteria of a phenotype of frailty as weight loss, exhaustion, low grip strength, slow gait, and low physical activity. The presence of three or more criteria was defined as frailty. Frailty is a major medical condition in the elderly population because it is the determinant of quality of life and longevity [5]. The mechanisms underlying frailty are linked with multifactorial factors since inflammatory, nutritional, vascular, and metabolic factors may be involved [7]. A previous study found that chronic inflammation in the elderly correlated with frailty [8]. High levels of Interleukin-6 (IL-6), Tumor necrosis factor-alpha (TNF-α), and C-reactive protein (CRP) were associated with poor function and mobility status, lower muscle strength and muscle mass, and frailty in the older individual. Recently, multivariable measures of inflammation provided an easier approach to track the progression of frailty over time [9,10].

The impact of frailty in the elderly is the reduced ability to carry out daily activities, loss of muscle strength and balance, high risk of adverse health outcomes including mortality, institutionalization, falls, and prolonged hospitalization [4,11,12]. Therefore, if pre-frailty is found before any other geriatric diseases in the elderly, it can delay or reverse frailty status and prevent adverse health outcomes.

Currently, there are many recommendations suggesting the types of intervention for frail older adults including nutritional, social support, cognitive, physical activity and exercise. A previous study reported that a combined supplement and exercise could improve physical performance [13]. A recent review also showed that the treatment of frailty combining physical exercise especially strength, balance, flexibility, and endurance delays the occurrence of vulnerable conditions and can improve the prospect of a return to normal for the vulnerable person [14]. In addition, previous studies on the exercise interventions in the elderly with multiple diagnoses or hospitalized patients recommended that the combination of strength training and aerobic training improved muscle mass, muscle strength, muscle function, and balance [15,16]. More recently, a previous study found that muscle strength and balance is a key component in exercise programs to reduce frail and fall risk scores in pre-frail older adults [17]. According to recommendations made by the American College of Sports Medicine (ACSM) and other literature, exercise interventions should consist of aerobic, strength, balance, and flexibility [18,19,20].

To the best of our knowledge, there are a few studies of multicomponent exercise training, which combine the center- and home-based for community-dwelling older adults with frailty and use an inflammatory biomarker as an outcome measure. Therefore, our study program focuses on strength, balance, and endurance, which promotes and improves physical performance and functional capacity in older adults. As such, we aimed to study the effects of a Multicomponent Exercise Program (MCEP) and usual care on frail community-dwelling older adults. The hypotheses were that the Multicomponent Exercise Program should improve physical performance including strength, balance, and endurance, reduce inflammation as measured with blood biomarkers including Interleukin-6 and C-reactive protein, reduce the frailty score assessed with the frailty phenotype and increase the health-related quality of life in older adults with frailty.

## 2. Materials and Methods 

### 2.1. Study Design and Participants

**Figure 1:**
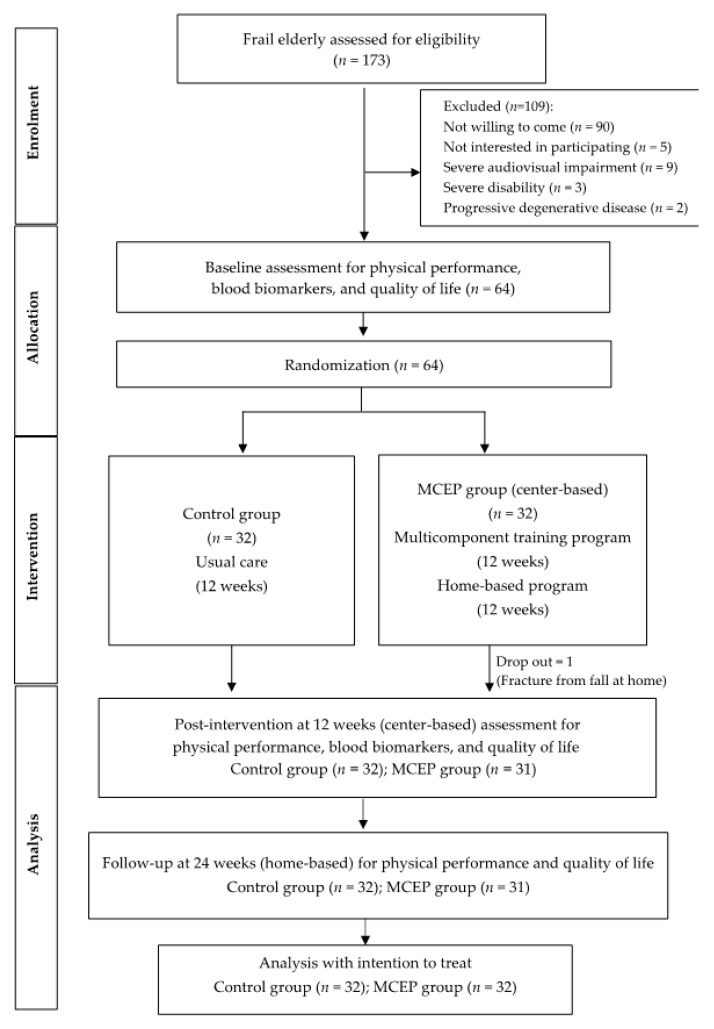
Flow chart of the study procedure.

The study was conducted from July to August 2018 in a Sub-district community in Phayao province, Northern Thailand. 173 frail elderly aged 65 years or older and had been identified as frailty according to Fried’s Frailty Phenotype [4] were recruited for eligibility. Exclusion criteria were progressive degenerative diseases, Mini-Mental State Examination-Thai version (MMST10) score less than 23 [21], severe audiovisual impairment, severe disability and were not willing to come. Thus, 109 participants were excluded; 90 not willing to come, 5 were not interested in participating, 9 had severe audiovisual impairment, 3 had severe disability, and 2 had progressive degenerative diseases. Therefore, 64 eligible participants were enrolled in the study and randomly allocated into two groups, a Multicomponent Exercise Program (MCEP) group; *n* = 32, and a control group; *n* = 32. Before the end of the trial, one participant in the MCEP group withdrew due to falls (not related to the study), resulting in a drop-out rate of 1.6%. The Consolidated Standards of Reporting Trial (CONSORT) flowchart that outlines the flow of participants through the study is shown in Figure 1.

The MCEP was a parallel-group, randomized controlled trial with a 12-week intervention (center-based) and 12-week follow up (home-based); assessment taking place at baseline, 12 weeks and 24 weeks by trained assessors who were blinded to the group allocation of the participant (Figure 1). The trial is registered with the Thai Clinical Trials Registry as ID: TCTR20180724003 (URL: http://www.clinicaltrials.in.th/index).

The sample size was calculated from a previous study of a multicomponent exercise program in frail nonagenarians, which consisted of muscle power training by using a manual dynamometer [22], with effect size 0.53 and 80% power at an alpha level of 0.01 and a dropout rate of 20%. Sixty-four participants were recruited and randomly assigned to the two groups; 32 in the MCEP group and 32 in the control group. The participants (mean age was 77.78 ± 7.24 years) were allocated with block randomization, where the sequence was generated in permuted blocks (8 blocks, 6 per block). At this step, we used the assisted double blinding technique to divide them into groups. All randomized participants met the eligibility criteria.

### 2.2. Intervention Program

The Multicomponent exercise program (MCEP), including aerobic training, resistance training, and balance training was tailored to participant ability by gradually increasing the intensity from moderate to high. It was of 60 min duration and took place over 3 days per week for 12 weeks directed by a qualified trainer at the health service center in the community and then 12 weeks following home-based exercises.

The MCEP consisted of songs and postures. A qualified trainer in exercise trained the volunteers three times per week for 1 month and then became the exercise leader to facilitate the intervention study. Both the exercise leader and physical therapists were present taking care of all participants throughout the study period. All participants performed the exercises together under observation and care from the leader and physical therapists. The participants of an MCEP group were divided into sub-groups, which had eight people in each group.

The MCEP was designed to improve strength, endurance, and balance for older adults, and was designed according to the Exercise Prescription for the Elderly [18,20,23,24] and the American College of Sports Medicine (ACSM) guidelines [19] Fitness Instructor Training Manual [25] as our program was for frail older adults. This modification was in accordance with that of previous studies [26,27,28]. The MCEP gradually increased in level of difficulty overtime (Table 1).

The sessions consisted of created motivation activity including interesting environments, relaxing songs, and the best practice model of this activity. The exercise program was classified into three components (aerobic training, resistance training, and balance training). Details of the exercises and their progression are described in Table 1. All participants always began with a warm-up (5−10 min) at HR < 40% maximal HR followed by aerobic training [28] while sitting on a chair, about 15 min, initially at 40% of maximum heart rate increasing progressively to 65%, involving leg marching, arm swing, tap, and clap, side bend, and arm raised. To ensure safety, the exercise intensity was controlled at 12−13 (somewhat hard) according to the Rate of Perceived Exertion (RPE) [29]. In the second exercise session, resistance training with a theraband was practiced after aerobic training. The intensity was set by the color of the theraband, (red, green, blue, and black (1−8 kg)). The participants were instructed to complete resistance training at a RPE of 12−13 (somewhat hard), while sitting on a chair. There were 10 exercise stations involving arm curl, backward arm press, hip flexor, hip extensor, hip adductor, hip abductor, knee flexor, knee extensor, ankle plantar flexor, and ankle dorsiflexor, which focused on the lower extremities. The exercise intensity was moderate to high with an exercise duration of 30 min. One-repetition maximum (1RM) voluntary strength measurements were performed at baseline. Initially, the intensity was set at 65% of the 1RM, and 1−2 sets of 6−8 repetitions of each exercise. At the end of the first month, the intensity was set at 85% to 100% of the initial 1RM, and 3 sets of 8−12 repetitions of each exercise. The third exercise component was balance training with 8 stations including a static and dynamic balance adapted from daily life and the Otago home exercise program [30]. This session took about 15 min. Balance stations included sit to stand, knee bends, backward walking, walking and turning around, sideways walking, heel-toe standing, heel-toe walking and one leg stand (Table 1).

A home-based exercise program, 60 min duration and took 3 days per week for 12 weeks, was assigned for the intervention group after they finished center-based exercise intervention. It consisted of the multicomponent exercise booklet with a description and safety advice. To assess their compliance, the subjects were asked to fill out a daily record after exercise. We made follow-up visits every week and provided assistance regarding the daily practice.

For the control group, usual care was done by the general practitioner and medical specialist that would be available to the older adults.

### 2.3. Outcome Measurements

Data were collected at baseline and after the 12th and 24th weeks of the intervention by the researcher assistants who were blinded to the group to which each participant had been assigned. The primary outcomes of physical performance including strength using handgrip dynamometer, balance using the Berg Balance Scale (BBS) and Timed Up and Go (TUG) tests; endurance using VO_2_max, blood biomarkers and frailty scores were assessed. Subjects also completed a questionnaire on demographic information, which included age, sex, health information, BMI, and the secondary outcome of Health-Related Quality of Life (HRQOL).

Muscle Strength was assessed by using grip strength (Dynamometer dynamometer Takei, T.K.K. 5401, Tokyo, Japan). Grip strength measurement is a valid and reliable tool for assessing grip strength in sarcopenia research [31]. The dominant hand of each participant was used for the test. The participants applied the dynamometer with maximum strength three times. The highest score was chosen to record.

The balance was tested using the BBS and TUG tests. The BBS consists of 14 items. Each item had a five-point (0−4), start at 0 to 4, and the total score was 56. If a score had less than 45 scores, then this increased their risk of falls [32]. For the TUG test, the participants sat on a standard 45 cm height chair, then had to get up and walk 3 m forward as fast as possible in comfort, turn around an obstacle, and return to sit on the chair again [33].

Endurance was assessed by VO_2_Max that was assessed by the 6-min walk test (indirect calculation method) [34]. In the test, the participants walked in a square for 6 min throughout the test. An assistive walking device was permitted if the participant needed it while their symptoms were recorded.

Serum interleukin-6 (IL-6) and C-reactive protein (CRP) levels were determined using the sandwich enzyme-linked immunosorbent assay (ELISA) method, with the human IL-6 ELISA MaxTM Set Deluxe Kits (BioLegend, San Diego, CA, USA) and the human CRP ELISA (Hycult Biotech, Uden, the Netherlands) commercial kits, respectively. The assay protocol was carried out according to manufacturers’ instructions. Briefly, for the quantification of IL-6, microtiter plate wells were coated with the rat monoclonal antibody specific to human IL-6. Subsequently, the standards and serum samples were added to the immobilized capture antibody, followed by a biotinylated rat anti-IL-6 monoclonal detecting antibody. This sandwich complex was visualized by adding horseradish peroxidase (HRP)-conjugated avidin followed by tetramethylbenzidine (TMB) substrate. The reaction was finally stopped by adding 1N HCl solution. The absorbance was read at 450 nm with an ELISA microplate reader (Bio-Rad, Hercules, CA, USA). For CRP measurement, the serum samples and standards were diluted using a specimen dilution buffer with 1:1000 and 1:100, respectively. These diluted samples and standards were then added into the anti-human CRP antibody-coated wells. The immune complex was detected by adding a peroxidase-conjugated antibody, followed by TMB substrate. The ELISA reaction was halted by the addition of sulfuric acid. The detection limits of both assays were 4 pg/mL for IL-6 and 5 ng/mL for CRP.

The frailty phenotype was measured based on five criteria. If the presence of 3, 4, or 5 criteria were defined as frail [4]. The frailty criteria were:

(a) Weight loss: self-reported unintentional weight loss > 4.5 kg in the previous year;

(b) Slow gait: assessed by 4.5 m fast walking test. The participants who had more than 6 s of the walking time were classified as slow gait;

(c) Weakness: assessed by a dynamometer (dynamometer Takei, T.K.K. 5401, Tokyo, Japan). The cut-off value for grip strength: male with 30 kg or less and female with 18 kg or less were classified as weakness;

(d) Exhaustion: evaluated by using self-reported exhaustion with two questions as follows: “I felt that everything I did was an effort” and “I could not get going”;

(e) Low physical activity: assessed by the Global Physical Activity Questionnaire (GPAQ) [35]. The cut-off value for males with less than 383 Kcals per week and females with less than 270 Kcals per week were classified as low physical activity.

HRQOL was assessed by the 36 items Health Survey (SF-36). The 36 items Health Survey (SF-36) consisted of the physical component summary (PCS) and the mental component summary (MCS). The generic health of SF-36 had eight categories as follows: physical functioning, role limitations due to physical problems, bodily pain, the general perception of health, vitality, social functioning, role limitations due to emotional problems, and mental health [36].

### 2.4. Statistical Analysis

All statistical analyses were performed using SPSS software, version 22.0 (IBM Corp., Armonk, NY, USA). The normality of the data was determined using the Shapiro–Wilk test and homoscedasticity of the variable was considered using Bartlett’s test. Independent sample t-tests for continuous data, and Chi-square test for categorical data were used to compare the demographic variables. All analyses were performed under an intention-to-treat approach. Two-way mixed-models analysis of variance (ANOVA) were conducted to determine significant main effects and interactions. The Bonferroni post-hoc test was used for multiple comparisons. The level of statistical significance was set at *p* < 0.05, two-sided.

### 2.5. Ethical Consideration

The trail was approved by the Research Ethics Committee of the Faculty of Medicine, Chiang Mai University, Thailand approved (Number: 273/2017)**.**

## 3. Results

### 3.1. Baseline Descriptive Data

The baseline characteristics of participants are presented in Table 2. The demographic characteristics of participants between the MCEP and the control groups had no significant differences at baseline. The MCEP group attended a center based program based on an average of 34 of the 36 exercise sessions (94.4%) and home-based programs on average 21 of the 36 exercise sessions (57.5%). No study-related injuries or falls were reported (data not shown).

### 3.2. Changes in Physical Performance During the 24-Weeks Intervention Period

**Figure 2:**
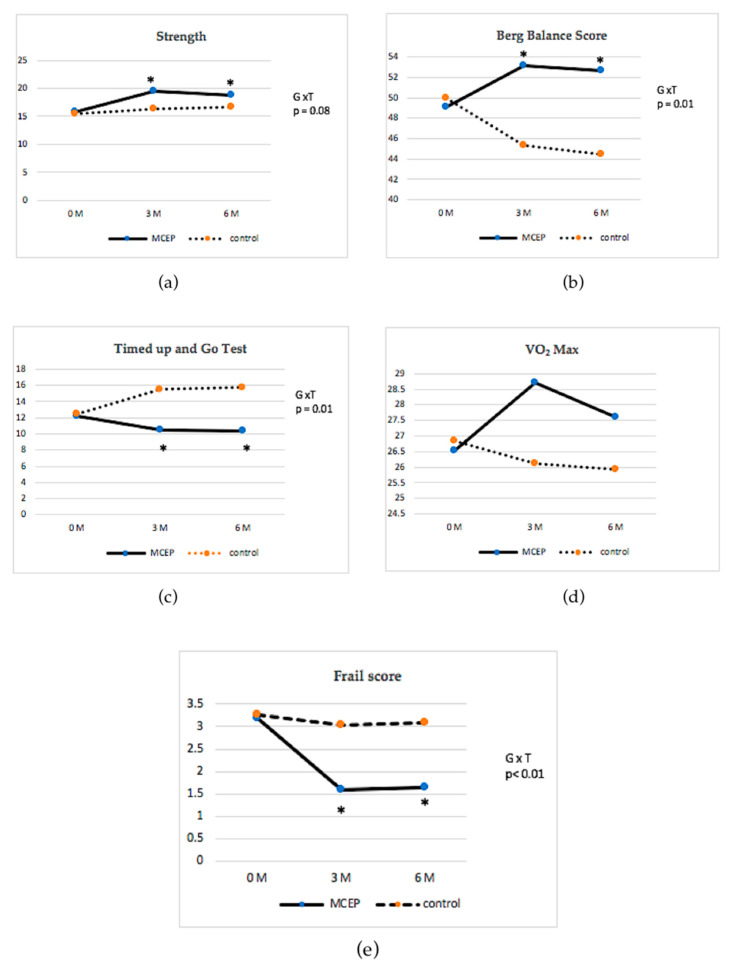
Change in physical performance from baseline to the end of the study between the intervention and control groups with interaction effects of group x time as follows: (**a**) change in strength; (**b**) change in Borg Balance Score; (**c**) change in Timed Up and Go test; (**d**) change in Vo_2_ Max; and (**e**) change in frailty score. Values are mean ± standard deviation. * = Significantly different at *p* < 0.05.

Baseline and post-intervention (12 and 24 weeks) primary outcome data are presented in Table 3 and Figure 2. Mixed model repeated measure ANOVA demonstrated significant interaction effects of group x time for balance variables as measured by Berg Balance Score (BBS) *(p* < 0.01) and Timed Up and Go (TUG) (*p* < 0.01). Post hoc analyses revealed that the MCEP group < showed significant improvements in the BBS (*p* < 0.01) and TUG (*p* < 0.01) when compared to the control group and their baseline after both 12 and 24 weeks of the intervention. In the case of strength and endurance variables, the MCEP group showed greater improvements in strength *(p* = 0.03) and VO_2_Max (*p* = 0.02) than the control group after 12 weeks of the intervention. However, there were no significant group x time interactions for these variables.

### 3.3. Changes in Frailty Score and HRQOL During the 24-Weeks Intervention Period

Baseline and post-intervention (12 and 24 weeks) secondary outcome data are presented in Table 3 and Figure 2. The mixed model repeated measure ANOVA revealed significant interaction effects of group x time for frailty score (*p* < 0.01). Within-group analyses showed that the MCEP group had significantly decreased frailty scores (*p* < 0.01) when compared with baseline after 12 and 24 weeks of the intervention. Furthermore, the MCEP group registered significantly improved quality of life as measured by SF-36 when compared with baseline after both 12 and 24 weeks of the intervention.

### 3.4. Changes in Blood Biomarker During the 12-Weeks Intervention Period 

Baseline and post-intervention (12 weeks) blood biomarkers are presented in Table 4 and Figure 2. The mixed model repeated measure ANOVA revealed significant interaction effects of group x time for IL-6 (*p* < 0.01) and CRP (*p* < 0.05). The MCEP group demonstrated significantly decreases in the IL-6 and CRP in comparison to the control group after 12 weeks (*p* < 0.05).

## 4. Discussion 

This study aimed to determine the effects of a multicomponent exercise program (MCEP) on frailty in older adults. To our knowledge, this was the first intervention trial that examined the effectiveness of the combined center-based and home-based multicomponent program in improving physical performance and reducing frailty, and reducing inflammatory biomarkers (IL-6 and CRP). This exercise program should be safe for older adults with frailty. The average rate of adherence at a center-based program was high (94.4%), home-based program was 57.5%, and no injuries were observed in our program. 

The main findings of this study showed that provision of an MCEP can improve all three physical performance tests including muscle strength, balance, and endurance after 12 weeks of training in frail older adults. Balance (BBC and TUG) and strength significantly differed from the baseline to both 12 weeks and 24 weeks (*p* < 0.001) (Table 3, Figure 2). The improvement of balance in the intervention group could be due to the joints that were stimulated by proprioceptors which promoted stability [37]. Balance improved physical function and the level of physical activity [14]. Previous studies suggested that the multicomponent exercise program improved the ability of gait, balance, and strength in the elderly, especially with frailty, and should consist of endurance, strength, and balance training [38,39,40]. Another reason that the balance was correlated positively with muscle strength.

Our study included strength and endurance training and was shown to be the most effective way to improve both neuromuscular and cardiorespiratory functions that related to the improvement of the balance score [41]. Moreover, muscle strength is an important component of the frailty phenotype. The increases in muscle strength which are a major component of physical frailty induced by our program, consistent with the results of previous studies showed that frail older people retain the capacity to adapt to high resistance training [42]. The statistically significant results for the strength measures could be due to the MCEP training being of moderate to high intensity and gradually increased every month. Recent studies show a strong link between high intensity training and the increases in strength [43,44].

In the endurance training aspect, we used chair aerobic exercise due to the need for care in dealing with frail older adults. A systematic review showed that chair-based exercise is a safe and suitable exercise for a vulnerable population who cannot participate safely in other exercise forms [28]. Our study showed the endurance level, as measured by VO_2_Max (indirect calculation from 6MWT) in an MCEP group, increased in scores but changed in VO_2_Max level, were not statistically significant. To prevent the cardiorespiratory decline observed during aging, endurance training periods ranging from 12 to 24 weeks with a weekly frequency of 3 to 5 times per week and a duration of exercise ranging from 30 to 60 min and intensity ranging from 50 to 85% of the maximal heart rate can significantly increase VO_2_Max [45]. The American College of Sports Medicine suggested that aerobic exercise training for increases in VO_2_Max should have sufficient intensity (60% of VO_2_Max for initial training), frequency, and length (3 days per week for 16 weeks) [24]. Additionally, the previous studies [26,27,28] supported applying the detail of this program. The progression of aerobic exercise level was assessed by the ability to do the exercise and the Rate of Perceived Exertion (12−13). However, our study needed to be particularly aware of safety for the participants to prevent adverse health outcomes from the intervention. Therefore, we designed short duration times in the endurance part of 20 min, 3 times per week for a 12-weeks period and a moderate intensity which increased the VO_2_Max level in due time. 

A trait of the aging process is low-grade chronic inflammation. The elevation of different cytokines (IL-6, TNF-α and CRP) is associated with poor function in mobility, frailty, disability, and mortality [8,46,47]. Chronic inflammation contributes to the increased risk of frailty, potentially mediated via neurodegeneration [48,49]. Moreover, inflammation plays an important role in the regulation of muscle turnover. Chronic inflammation represents a common trait of several pathophysiological processes leading to muscle loss [50]. Therefore, pro-inflammatory molecules such as IL-6 and CRP are suitable biomarkers that provide useful information for the early identification of frailty. Findings in this study indicated that the level of inflammatory biomarkers of frailty, IL-6 and CRP significantly decreased when compared to the control group. Moreover, the crude endpoints level of IL-6 and CRP of MCEP were reduced when compared between baseline and post-intervention, however, this was not significant. In contrast, the level of IL-6 and CRP did not change when compare between baseline and postintervention. This result agreed with several studies indicating that increasing levels of physical activity are associated with reduced levels of proinflammatory cytokine such as IL-6, TNF-α, and CRP [7,10,51].

We found that frailty was reversed in the MCEP group after the exercise training program. This was evidenced by changes in the mean frailty score from baseline to 12 weeks and 24 weeks, 3.18 to 1.59 and 1.65 scores, respectively. Whereas there was no evidence of reversed frailty in the control group after the 24 weeks period. Therefore, it is likely that the improvement in components such as strength and balance leads to an increase in physical activity, reducing the degree of frailty. These findings are supported by three other studies. One study showed the effects of a 12-weeks exercise training program on physical function in frail elderly to improvement in muscle strength and reversal of the frail condition [52]. The other two studies showed that multicomponent exercise intervention including endurance, strength, coordination, balance, and flexibility exercises reverse frailty and improve physical function in older adults with frailty [26,53].

Moreover, other systematic reviews agreed that the multicomponent training, with long duration, 30–45 min per session, and 3 times per week, is the most common protocol for exercise for the frail elderly. Specifically, multicomponent exercise comprising resistance and/or balance and/or flexibility exercises are most effective with frail older adults [14,20] All results reported the effects of balance, muscle strength, and functional ability significantly. Exercise programs should especially consider the effects on physical performance by gender, as females have more disadvantages than males [54,55]. In our study, the intervention characteristics were as follows: 12 weeks center-based and 12 weeks home-based, 3 days per week, and 60 min per session. In the center-based intervention, at 12 weeks post intervention, the MCEP group was more effective than a control group because the MCEP in the center-based intervention has lead trainers which advice and provide feedback to participants. As for t 24 weeks home-based follow up, the MCEP group maintained improvements, only in strength, balance, frailty score, and physical component summary in quality of life. The reason may be that home-based sessions had low adherence (57.5%), which may indicate the reduction in a participant’s motivation. Thus, home-based participants needed a strategy to increase adherence. A previous study suggested that supervised exercise programs tend to motivate participants more efficiently [56]. Previous studies demonstrated that frail older adults could improve physical performance, strength, and Vo_2_Max after practiced resistance and aerobic exercise [20,41]. However, with less training duration and intensity, this study demonstrated a trend toward grip strength and Vo_2_Max improvement in the MCEP group. Together, it is likely that the lack of significant improvement in grip strength and Vo_2_Max was due to the suboptimal dose of exercise training. However, multicomponent exercise training with supervision is the medicinal therapy that can prevent frailty as well as reverse it.

Health-Related Quality of life (HRQOL), as measured by SF-36 in the MCEP group was significant in the physical component summary (PCS) at week 12. These results were in line with another study, which reported that exercise programs including muscle and balance training, increase physical activity and improve physical and life functions such as muscle strength, walking ability, and activities of daily living which relates to the HRQOL for frail elderly [57]. In the frail, the physical dimensions of quality of life were the most affected [58]. In our exercise program, a focus on physical performance which improves strength and balance were the reason behind the increase in physical component summary scores.

Participants in the control group who were asked to maintain normal daily-living habits and usual care for the duration of the 24-weeks study, showed no significant changes in their week 12 and week 24 measures with respect to baseline measures.

In summary, our findings indicate that an exercise program with a supervised moderate to high-intensity training is effective and safe for community-dwelling older adults with frailty. MCEP improves physical performance, especially balance, to a greater extent than usual care. Future studies should be focused on the long-term effect of the MCEP with different and more severe levels of frailty and its utility in individuals, and may specify the selection of equal numbers of both genders for exercise intervention. Additional strengths of the study are the generalizability to frail older people and aged care health service settings. There were some limitations in this study. For example, it was not possible to blind participants and clinicians/ trainers for this type of exercise program. Although outcome assessors were blinded to the groupings, some participants might have inadvertently disclosed their treatment status. Another limitation was that this study was of a relatively short time duration. A longer duration of study is needed in order to determine effects especially on the levels of the psychological component. This study was not classified according to frailty severity, thus an optimal training dose for individual training is yet to be determined.

## 5. Conclusions

In conclusion, this study demonstrated that this multicomponent exercise program improves physical performance especially balance, and quality of life, delays frailty and decreases inflammation in frail older adults. Importantly, MCEP is safe and suitable for frail older adults to practice at home.

## Figures and Tables

**Table 1:** Multicomponent exercise program protocol.

Exercise Training	Description	Duration	Intensity Progression
Chair Aerobic Training	(i) seated marching, (ii) leg marching, (iii) arm swing, (iv) tap and clap, (v) side bend, and (vi) arm raised	10−20 min	month 1: 10 min month 2: 15 min month 3: 20 min
Resistance Training with Theraband	(i) arm curl, (ii) backward arm press, (iii) hip flexor, (iv) hip extensor, (v) hip adductor, (vi) hip abductor, (vii) knee flexor, (viii) knee extensor, (ix) ankle plantar flexor, and (x) ankle dorsiflexor	25−30 min	month 1, Reps: 8 × 2 set, intensity: 65% of the 1RM month 2, Reps: 10 × 3 set, intensity: 75% of the 1RM month 3, Reps: 12 × 3 set, intensity: 85−90% of the 1RM (Intensity was set by the color of the theraband.)
Balance Training	(i) sit to stand, (ii) knee bends, (iii) backwards walking, (iv) walking and turning around, (v) sideways walking, and (vi) heel toe standing (vii) heel toe walking (viii) one leg stand	10 min	Month 1: two hands support month 2: one hand support month 3: no hand support

**Table 2:** Baseline characteristics of participants.

Characteristics	Control Group (*n* = 32)	MCEP Group (*n* = 32)	*p*-Value
Age (y), mean ± SD	78.87 ± 1.32	76.68 ± 1.14	0.64
Sex, *n* (%)			
Female	16 (50.0)	23 (71.9)	0.06
Male	16 (50.0)	9 (28.1)	
Number of Comorbidities, mean ± SD	1.25 ± 0.76	1.09 ± 0.96	0.35
Number of Types of Medication, mean ± SD	2.62 (1.91)	2.28 ± 1.97	0.67
Self-health Rating, *n* (%)			
Excellent	4 (12.5)	6 (18.8)	0.61
Good	19 (59.4)	16 (50.0)	
Fair / poor	9 (28.1)	10 (31.2)	
BMI, mean ± SD	21.28 (0.69)	21.37 (0.68)	0.84
HRQOL Score (range, 0−100), mean ± SD	60.77 (16.84)	61.45 (13.3)	0.27
Frailty Score, mean ± SD	3.25 (0.50)	3.18 (0.39)	0.58
Frailty Criteria, *n* (%)			
Weight Loss	8 (25.0)	6 (18.8)	0.76
Low Grip Strength	31 (96.9)	29 (90.6)	0.61
Low Walking Speed	25 (78.1)	24 (75)	1.00
Exhaustion	15 (46.9)	17 (53.1)	0.84
Low Physical Activity	29 (90.6)	32 (100.0)	0.27

MCEP = Multicomponent exercise program, BMI = Body Mass Index, HRQOL = Health-related quality of life. Statistical significance at *p* < 0.05 using Independent Sample t-test for continuous data, and Chi-square test for categorical data.

**Table 3:** Comparisons of outcome between groups in physical performance, frailty score and HRQOL at baseline, 12 and 24 weeks.

Parameters	Control Group (*n* = 32)			MCEP Group (*n* = 32)			Group × Time ^#^
	Baseline	12-Weeks	24-Weeks	Baseline	12-Weeks	24-Weeks	*p*
**Physical Performance**							
Strength (Handgrip (kg))	15.50 ± 6.47	16.28 ± 7.00	16.70 ± 8.05	15.71 ± 6.21	19.56 ± 5.27 *^,†^	18.84 ± 5.01 ^†^	0.08
Berg Balance Score	49.96 ± 4.40	45.34 ± 8.65	44.46 ± 9.52 ^†^	49.12 ± 3.58	53.12 ± 3.16 *^,†^	52.68 ± 3.49 *^,†^	<0.01
TUG (sec)	12.43 ± 5.04	15.57 ± 7.65 ^†^	15.75 ± 6.96 ^†^	12.21 ± 2.26	10.48 ± 2.16 *^,†^	10.33 ± 2.91 *^,†^	<0.01
VO_2_Max	26.85 ± 4.57	26.12 ± 4.60	25.92 ± 4.07	26.53 ± 3.14	28.69 ± 4.39 *	27.59 ± 4.14	0.07
**Frailty Score**	3.25 ± 0.50	3.03 ± 1.20	3.09 ± 0.92	3.18 ± 0.39	1.59 ± 0.83 *^,†^	1.65 ± 0.86 *^,†^	<0.01
**HRQOL; SF-36**							
Overall	61.28 ± 16.17	60.49 ± 21.63	56.69± 13.34	61.90 ± 13.23	61.79 ± 20.92	59.56 ± 14.00	0.58
PCS	53.56 ± 17.83	52.53 ± 20.43	52.82 ± 20.98	53.38 ± 14.28	64.88 ± 22.96 ^†^	60.78 ± 22.5	0.17
MCS	73.88 ± 17.41	70.68 ± 21.94	69.36 ± 20.28	71.11 ± 15.87	74.14 ± 21.79	72.81 ± 21.71	0.25

All data are expressed as means ± SD; ^#^ Analysis of two-way repeated-measures ANOVA; * = Significant difference between groups at *p* < 0.05; ^†^ = Significant difference between baseline and post-intervention at *p* < 0.05; HRQOL = Health Related Quality of Life; SF-36 = 36-Item Short Form Survey; PCS = Physical Component Summary; MCS = Mental Component Summary.

**Table 4:** Comparisons of outcome between groups in blood biomarker at baseline and 12 weeks.

Blood Biomarker	Control Groupleft (*n* = 32)		MCEP Groupleft (*n* = 32)		Group × Time ^#^
	Baseline	12-Weeks	Baseline	12-Weeks	*p*
IL-6	11.93 ± 17.56	11.04 ± 8.93	10.15 ± 6.25	8.16 ± 8.58 ^†^	0.005
CRP	3.97± 5.82	4.60 ± 6.91	3.83 ± 5.13	2.49 ± 4.46 ^†^	0.022

All data are expressed as means ± SD; # Analysis of two-way repeated-measures ANOVA. ^†^ = Significant difference between baseline and post-intervention at *p* < 0.05.
